# Can Dietary n-3 Polyunsaturated Fatty Acids Affect Apelin and Resolvin in Testis and Sperm of Male Rabbits?

**DOI:** 10.3390/molecules28176188

**Published:** 2023-08-22

**Authors:** Simona Mattioli, Elena Moretti, Cesare Castellini, Cinzia Signorini, Roberta Corsaro, Elisa Angelucci, Giulia Collodel

**Affiliations:** 1 Department of Agricultural, Environmental, and Food Science, University of Perugia, Borgo XX Giugno 74, 06123 Perugia, Italy; simona.mattioli@unipg.it (S.M.); cesare.castellini@unipg.it (C.C.); elisa.angelucci@unipg.it (E.A.); 2 Department of Molecular and Developmental Medicine, University of Siena, Policlinico Santa Maria alle Scotte, Viale Bracci 14, 53100 Siena, Italy; cinzia.signorini@unisi.it (C.S.); roberta.corsaro@student.unisi.it (R.C.); giulia.collodel@unisi.it (G.C.)

**Keywords:** angiotensin-like-receptor 1, apelin, diet, ejaculated sperm, oxidative stress, PUFA, resolvins, ryanodine receptor, testes

## Abstract

Apelin and other novel adipokines have been associated with normal and pathological reproductive conditions in humans and animals. In this paper, we used a rabbit model to investigate if apelin and resolvin (RvD1) in testis and sperm are associated with the oxidative status of semen and serum testosterone of rabbits fed different diets enriched with flaxseed (alpha-linolenic acid, ALA) or with fish oil (eicosapentaenoic acid, EPA, docosapentaenoic acid, DPAn-3, and docosahexaenoic acid, DHA). Apelin and RvD1 were detected by ELISA and apelin and the apelin receptor by immunofluorescence. Increased levels of apelin in testes from both enriched diets were shown, particularly in the interstitial tissue of the FLAX group. The FLAX diet enhanced serum testosterone, and both enriched diets showed higher levels of malondialdehyde and RvD1 in the testis. In ejaculated sperm, apelin and its receptor were localized in the entire tail of the control and both treated groups. The ryanodine receptor was investigated in rabbit testis; the fluorescent signal was increased in mature elongated spermatids of the FLAX group. In conclusion, this data seems to indicate that FLAX increases the amount of apelin in testis, suggesting an involvement of this adipokine in male reproduction and probably a role in the resolution of the inflammatory status.

## 1. Introduction

The lipid profile of a diet deeply affects the reproductive functions of humans and other animal species [1]. The main effects are related to several changes in the lipid profile of germ cells and reproductive tissues, and to modification of hormonal assets and the cascade of some lipid-derived molecules (isoprostanes, sterols, etc.) [2]. Oxidation and inflammation are relevant endpoints of this complex interaction among dietary lipids and derived molecules since dietary lipids modulate the fatty acids profile of tissues and, thus, their oxidative onset [3].

Supplemental dietary n-3 PUFA improved sperm motion traits and resulted in an enrichment of membrane fatty acid in the sperm and testes of the rabbits [2]. Testes showed high gene expression of enzymes involved in PUFA synthesis, as Δ6 desaturase (FADS2), elongase (ELOVL)2, and ELOVL5, and the low expression of Δ5 desaturase (FADS1). Intermediate metabolites, enzymes, and final products were found differently in Leydig, Sertoli, and germinal cells [4].

In recent studies, adipokines, such as adiponectin, chemerin, visfatin, resistin, and omentin, have been associated with normal and pathological reproductive conditions in humans and animals [5,6,7].

Apelin is an endogenous peptide that is expressed in the brain, placenta, heart, lungs, kidneys, pancreas, testis, prostate, and adipose tissues. It has been suggested that apelin affects energy metabolism [8] and the release of inflammatory mediators [9]. Apelin also inhibits the release of reactive oxygen species (ROS) in adipocytes, promotes the expression of antioxidant enzymes [10], and seems to play a protective role in the progression of lymphatic tumors. Moreover, apelin is locally synthesized in the hypothalamus, pituitary gland, ovaries, and testes of many species, showing autocrine and/or paracrine effects [11]. The receptor of apelin, called angiotensin-like-receptor 1 (APJ), and the apelinergic system are expressed in several tissues of the brain, spleen, placenta, heart, liver, intestine, prostate, thymus, ovary, lung, kidney, stomach, adipose tissue, and testis, where many pleiotropic effects are observed.

Moustafa [12] observed that in rats, the administration of n-3 or n-6 fatty acids impaired steroidogenesis but improved the antioxidant and anti-inflammatory status of the reproductive system via modulation of adipokines in the testicles. Das et al. [13] indicated that apelin may be a signal during the early postnatal stage for the regulation of germ cell proliferation, apoptosis, and expression of androgen receptors. Accordingly, it seems that the diet and the availability of some nutrients (e.g., fatty acids) have a relevant role in sperm physiology and in the homeostasis of the apelinergic system [14].

Recently apelin/APJ-R system has been localized in human spermatozoa and testicular tissue and is probably involved in human fertility [15].

At the same time, it appears that other molecules derived from omega-3 fatty acids (eicosapentaenoic acid, EPA, docosapentaenoic acid, DPAn-3, and docosahexaenoic acid, DHA), like resolvin (Rv)D1, play a major role in promoting the restoration of normal cellular function following an inflammation occurring after tissue injury [16,17].

RvD1 amount was positively correlated with F_2_-IsoPs and reduced sperm quality, and it increased along with other markers of oxidative stress and inflammation as fatty acids content and clinical biomarkers [18].

In this paper, we used the rabbit as a model to investigate if the apelinergic system (apelin and APJ) in sperm and testis and RvD1 may be associated with the fatty acids profile and the oxidative status semen and serum testosterone of rabbits fed different diets enriched with flaxseed, which has a very high alpha-linolenic acid (ALA) level, or with fish oil that directly supplies ALA derivatives (EPA, DHA, and DPA). The possible involvement of the ryanodine receptor (RyR), a lipid mediator of resolution of inflammation, the major cellular mediator of induced calcium release, was also detected.

## 2. Results

Testosterone level, sperm parameters and fatty acid profile of sperm, and oxidative status are shown in Table 1.

The FLAX group showed the highest testosterone concentration, whereas the FISH group showed the lowest level (3.63 vs. 4.60 and 2.82 pg/mL, respectively, in the control, FLAX, and FISH groups).

The sperm motility and curvilinear velocity (VCL) increased in the rabbits fed FLAX and FISH diets.

Sperm MDA, as well as n-3 PUFA and n-3 VLCP, were higher in enriched dietary groups compared to the control. In the latter groups, n-6 PUFA and n-6 VLCP resulted in significantly lower values with respect to the control (Table 1).

Apelin and RvD1, as well as MDA level, n-3 and n-6 PUFA, and n-3 and n-6 VLCP in testis, are shown in Table 2. Apelin resulted higher in the FLAX group compared to the control and FISH groups. RvD1 showed higher values in both enriched dietary groups with respect to that found in the control. MDA, n-3 PUFA, and n-3 VLCP were higher in the FLAX and FISH groups compared to the control, while n-6 PUFA and n-6 LCP resulted in lower values (Table 2).

The correlation among the previous traits is shown in Table 3 and Supplementary Table S1. A high positive (*p* > 0.01) correlation was recorded between apelin, RvD1, and lipid oxidation in the testis (MDA), whereas only the RvD1 was positively correlated with the sperm lipid oxidation (MDA). RvD1 also showed a positive correlation with the sperm kinetic parameters (VCL and motility), whereas the apelin was positively correlated with serum testosterone (T, *p* < 0.01) and VCL (*p* < 0.05).

n-3 PUFA in the testis showed a higher positive correlation with these latter proteins, whereas the sperm n-3 PUFA was only correlated with RvD1 (*p* < 0.01). On the contrary, n-6 PUFA was negatively correlated only with the RvD1 in both testis and sperm (*p* < 0.01). Testis and sperm lipid oxidation exhibited a strong correlation with the kinetic traits of sperm. As expected, n-6 and n-3 PUFA were inversely correlated both in the testis and sperm.

The n-3 intake (estimated considering that the feed intake was no different between groups, ~150 g/d) was correlated with almost all the parameters (positive with apelin, RvD1, MDA, T, VCL, Motility, and n-3 testis profile; negative with n-6 PUFA of sperm and testis) except n-3 PUFA of sperm, whereas n-6 intake was significantly correlated with apelin, testis MDA, and T.

**Figure 1:**
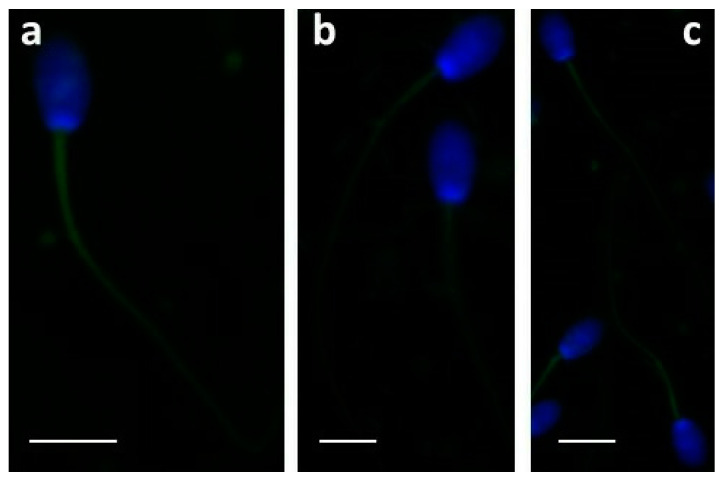
Ejaculated spermatozoa of the control (**a**) and FLAX (**b**) rabbits incubated with anti-apelin antibody. A fluorescent label was detected in all samples along the sperm tail (**a**,**b**). The APJ receptor was localized in the entire tail (**c**) in all the samples. Bars: 5 μm.

Immunofluorescence performed on ejaculated sperm showed that apelin was located on the entire tail (Figure 1a,b) of the sample from the control (Figure 1a) and enriched dietary groups (e.g., FLAX; Figure 1b). The APJ receptor was also detected in the tail in all the samples (control and both enriched diets) (Figure 1c).

**Figure 2:**
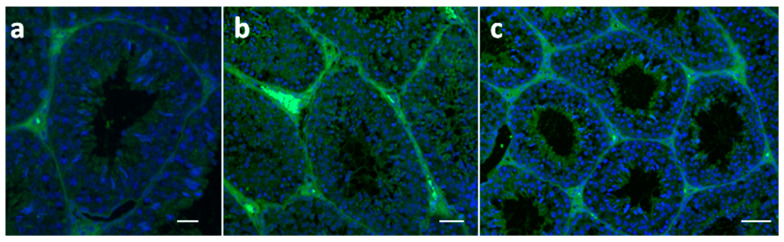
UV micrographs of rabbit testicular tissue treated with anti-apelin antibody from control (**a**) and enriched diets ((**b**) FLAX; (**c**) FISH). A weak fluorescent signal is present in the interstitial tissue of controls and FISH diets (**a**,**c**), respectively). In (**b**), high labeling intensity in the interstitial tissue is evident. In (**b**,**c**), spermatids are labeled. Bars: 50 μm.

As shown in Figure 2, apelin was strongly expressed in the interstitial tissue of the testis from the FLAX (Figure 2b) compared with the control (Figure 2a) and FISH groups (Figure 2c). Spermatids were labeled in both enriched dietary groups. As in sperm, the localization of the APJ was similar to what showed for apelin.

**Figure 3:**
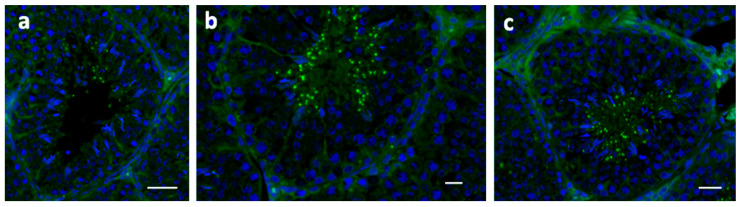
Immunolocalization of ryanodine receptors in rabbit testicular tissue from the control (**a**,**b**) FLAX, and (**c**) FISH groups. A dotted fluorescent signal is present in the seminiferous tubules in a reduced number of spermatids (**a**); the number of spots appears increased in (**b**,**c**), and strongly present in b, in particular. A faint localization in the interstitial tissue is shown (**b**,**c**). Bars: 50 μm.

Finally, the amount and distribution of ryanodine receptors were investigated in the testes. In the testes of the control rabbits, a faint label was observed in interstitial cells, and fluorescent spots were detected in a few numbers of spermatids (Figure 3a). A weak presence of the signal in samples from rabbits fed both enriched diets was observed in the interstitial tissue, and a high-dotted localization was detected in seminiferous tubules (Figure 3b,c). In particular, the fluorescence was increased in mature elongated spermatids of the FLAX group (Figure 3b).

## 3. Discussion

In this study, the possible role of apelin and its receptors, RvD1, and ryanodine receptors were investigated in the reproductive system and mature sperm of rabbits fed different diets enriched with ALA (FLAX group), or VLCP (FISH group). Rabbit represents a good animal model for studying sperm modifications due to the diet of other stressors (infection, inflammation, etc.) [19,20,21]. 

It is widely known that some adipokines, such as apelin, can regulate male and female reproduction according to the energy balance of the body. Chemerin, apelin, resistin, and visfatin are expressed in the ovaries of various animal species; however, the role of apelin in male reproduction has not yet been clarified [22]. Moretti et al. [15] detected increased levels of apelin and IL-1β concentrations in patients’ samples with varicocele and infections.

It has been reported that diets rich in n-3 fatty acids and low in saturated fatty acids and trans-fatty acids, if adequately protected with antioxidants (vitamins E, C, D, β-carotene, selenium, zinc, folate), are positively associated with sperm quality [2,23]. Different dietary sources of n-3 polyunsaturated fatty acids modify the lipid plasma membrane of the somatic and germ cells of the rabbit testis, even if they do not change the spermatogenesis and/or the ultrastructure [2]. This fact was confirmed in this study, where spermatozoa from rabbits of FLAX and FISH groups displayed increased progressive motility.

In this paper, ELISA and immunofluorescence analysis in the testes from both enriched diets showed that apelin was widely expressed, particularly in the interstitial testis of the FLAX group, in agreement with other authors that have reported high intakes of PUFA and VLCP [24]. Moreover, spermatids appeared labeled in both enriched dietary groups. 

Apelin and APJ were detected in the entire tail of ejaculated sperm in the control and both treated groups. In other cell models, apelin can reduce mitochondrial ROS-triggered oxidative damage [25], mitochondria apoptosis, and inflammatory responses stimulated by NF-κB and NLRP3 inflammasome [26]. Overproduction of ROS has been linked to sperm damage and male infertility. There are many pathological conditions, including varicocele, tobacco usage, alcohol, obesity/metabolic syndrome, leukocytospermia, and sexually transmitted infections [27], which enhance the production of radicals.

Recently, in mouse, apelin and its receptor were localized in the Leydig and germ cells, where they can influence testis steroidogenesis [13]. It has been reported that, in rats, intraperitoneal administration of apelin resulted in a decrease of serum testosterone, luteinizing, and follicle-stimulating hormone [28]. In the canine testis, apelin was detected in spermatids and mature sperm, and the APJ receptor was also detected in the cytoplasm of Leydig cells [29].

Exogenous treatment with other adipokines, as well as adiponectin, positively influenced testicular mass, insulin receptor expression, and testosterone synthesis in the testis of aged mice [30].

FLAX diet increased serum testosterone, and both the enriched diets enlarged MDA and RvD1 in the testis. Previously, Castellini et al. [2] showed persistent higher blood testosterone in rabbits fed a three-month FLAX diet. 

Different authors [31,32,33] suggested that dietary flaxseed, being one of the richest sources of phytoestrogens (lignans), may increase testosterone hormone secretion by modulating blood cholesterol; indeed, lignans are able to reduce blood cholesterol (mainly on low-density lipoprotein, LDL) competing with the cholesterol anabolism of liver and favoring the sterol-hormone one [34]. Furthermore, the flaxseed phytoestrogens possess, to a greater or lesser extent, functional similarity to the 17β-estradiol, thus, binding the same receptors of estrogen hormones (α and β-estrogen receptors) [35]. 

**Figure 4:**
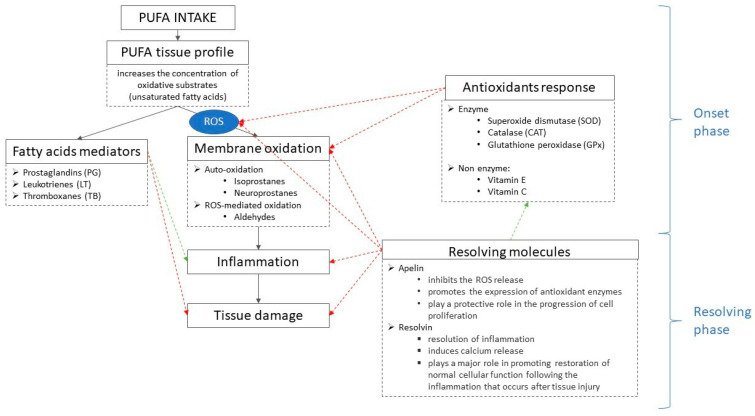
Tentative scheme of action of antioxidants and resolving molecules following high dietary PUFA intake. Solid line = mechanism flow; dotted line (arrows and squares) = molecule activity; red line = reducing activity; green line = enhancing activity. PUFA: polyunsaturated fatty acids; ROS: reactive oxygen species.

Serum testosterone, oxidation, and the amount of n-3 PUFA in the testis were positively correlated with apelin and RvD1. RvD1 is a member of the specialized pro-resolving lipid mediator family derived from LC, and consequently, it increased the lipid oxidation (testis and sperm MDA) induced by the higher PUFA concentration of enriched diets. However, the reproductive apparatus was able to counteract this higher oxidative thrust since RvD1 was correlated with sperm motility and VCL, suggesting that its activation improves sperm kinetic, probably operating an anti-inflammatory/antioxidant action on reproductive tissues, as hypothesized hereinafter (Figure 4).

In humans, the RvD1 amount was increased in patients with leukocytospermia, varicocele, and idiopathic infertility compared to fertile men and with other markers of oxidative stress and inflammation, such as fatty acids content and clinical biomarkers suggesting a possible role for a diagnosis of inflammatory status and a subsequent appropriate therapeutic approach [18].

Sperm motility is influenced by the lipid composition of the plasma membrane, largely determined by VLCP that ameliorates the flexibility of cells. The lipid bilayer of the rabbit sperm membrane consists mainly of cholesterol and phospholipids [36], which contain about 43% VLCP with more than 20 carbon atoms, with DPA n-6 being the most representative [4]. The VLCP in the sperm membrane is derived from linoleic acid (C18:2n-6; LA) and ALA resulting, in turn, from the dietary intake and are converted into their derivatives by the liver, or to a lesser extent, by testicular cells [4]. Furthermore, the lipid bilayers are constituted by different sterols (cholesterol and desmosterol, a cholesterol precursor), which, together with the phospholipids, modulate the membrane fluidity, sperm capacitation, and acrosome reaction [23]. Nordgren et al. [37] suggested that higher n-3 PUFA intake provides more substrate to produce pro-resolving lipid mediators during high-risk pregnancy/delivery conditions. Zirpoli et al. [38] observed that acute or chronic administration of n-3 PUFA enhanced RvD1, eliciting cardio- and neuroprotection through the activation of several interrelated anti-inflammatory pathways. 

Probably, increasing PUFA intake activates a complex mechanism consisting in triggering the cascade of several molecules, as follows: PUFA intake > PUFA tissues > oxidative thrust > oxidative markers (e.g., MDA, Isoprostanes) > antioxidants response (enzymatic and non-enzymatic) and resolving molecules (i.e., apelin and resolvin) = inflammation controlling or solving (Figure 4).

Indeed, it is reported that resolvins were enzymatically produced by oxidized VLCP metabolites; in particular, the RvD1 comes from DHA oxidation [39,40] in response to inflammation. There are two phases in acute inflammation: the onset phase and the resolving phase [39]. During the onset phase, fatty acids-mediators such as prostaglandins (PG) and leukotrienes (LT) operate as pro-inflammatory mediators, whereas after tissue injury or trauma, some specific mediators (i.e., PG-E2 and LT-B4) drives the neutrophils infiltration into the injured tissue, and remove dead cells. After that, it initiates the resolving phase, where pro-resolving lipid mediators act against acute inflammation with a mechanism that is not yet well defined.

PUFA intake increases the PUFA content of tissues, inducing, on the one hand, the oxidative thrust ROS-mediated (malondialdehyde MDA and isoprostanes) and from the other, the fatty acid-mediators release (prostaglandins PG, leukotrienes LT, and thromboxanes TB). The increase of fatty acid mediators modulates tissue inflammation (onset phase). Contemporarily, the antioxidant’s defense (enzymatic and non-enzymatic) acts against oxidation in the onset phase, followed by resolving molecules activation (resolving phase), which reduces ROS generation and tissue inflammation.

As previously reported, in physiological conditions, a higher intra-testicular expression of the apelinergic system in the mouse testes was associated with a reduction in testosterone secretion [13]; conversely, in this research, apelin was negatively correlated with the serum testosterone in the FLAX diet (Supplementary Table S1), probably due to phytoestrogens competition, as previously stated.

Moreover, higher levels of apelin in the testis of the FLAX group are associated with increased oxidation in the testis and sperm. These results underline that the number of antioxidants in the diet (i.e., α-tocopherol), even if considered supranutritional (50 vs. 200 mg/kg biblio), is not sufficient to balance the oxidation thrust induced by lipoperoxidation of VLCP. Compensatory mechanisms have probably been activated with possible effects on the reproductive system [3]. For example, the effect of vitamin E is partially compensated by the activation of enzymes (i.e., cytochrome p450) and by some other mechanism studied in this experiment (Figure 4), namely the increase of apelin and RvD1 for reducing the oxidation and/or its deleterious effect on cells of reproductive apparatus [41]. 

It is reported that in human neuroblastoma SH-SY5Y cells, apelin reduced calcium release, caspase-3, and cytochrome c, preventing apoptosis, oxidative stress, and mitochondrial toxicity [42]. 

The resolution of inflammatory processes may be also modulated by calcium channels. Therefore, finally, the presence of ryanodine receptors (RyRs) was investigated in the testes of rabbits. RyRs are intracellular calcium release channels that are highly expressed in striated muscles and neurons but are also detected in several non-excitable cells [43]. Spermatogenic cells express transcripts for all three RyR isoforms. However, there is no consensus regarding the presence and exact localization of RyRs in mature sperm [44]. This receptor was localized in the mitochondrial helix or in the entire sperm tail and may be involved in the capacitation process [45]. It is known that the atrial activity of the Ca^2+^/calmodulin-dependent protein kinase II (CaMKII) is higher during sepsis and causes hyperphosphorylation of cardiac ryanodine receptor 2 (RYR2) channels preventing neuronal excitotoxicity, smooth muscle relaxation, vasodilation, and immunomodulation [46]. RvD1 can reduce the pro-inflammatory phenotype of microglia and enhance phagocytosis of Aβ by microglia of AD patients and ameliorates the decline of phagocytosis of FAM-Aβ through binding to different receptors. The in vitro effects were concentration-dependent on MAPK, PI3K, and calcium signaling pathways [47].

On the other side, it has been recently reported that apelin influences the expression of mitochondrial calcium uniporter, which increases mitochondrial calcium uptake [48], suggesting a relationship between apelin levels and calcium that may have a fundamental role not only in inflammation resolution but in sperm physiology and fertilization ability (i.e., spermatozoa capacitation). 

## 4. Materials and Methods

### 4.1. Animals and Experimental Design

Fifteen New Zealand White male rabbits were trained for semen collection with an artificial vagina (50 days), then, they were divided into three homogeneous groups (5/group): the control group was fed ad libitum with the standard diet, the FLAX group was fed a standard diet, which was supplemented with 10% of extruded flaxseed, the FISH group was fed a standard diet, which contained 3.5% of fish oil (Nordic Naturals Omega-3^®^, Table 4). The dietary protocol involved 60 days. Animals were handled as reported in Boiti et al. [49]. The feed intake was registered weekly to calculate the approximate intake of n-3 and n-3 VLCP. Semen samples were evaluated at the beginning and at the end of the protocol. This study was conducted in accordance with the Guiding Principles in the Use of Animals and approved by the Animal Ethics Monitoring Committee of the University of Siena (CEL AOUS; authorization no. 265/2018-PR, ISOPRO 7DF19.23). 

Blood samples drawn from the marginal ear vein were collected in tubes containing Na_2_-EDTA and centrifuged at 5000× *g* for 15 min at 4 °C. Serum was obtained from blood samples coagulated at room temperature for 2 h, and then the collection tubes were rimmed and refrigerated at 4 °C for 24 h before analysis.

At the end of the trial (110 days), the rabbits were killed, and their testes were accurately removed; a part was fixed for immunofluorescence, and another part was stored at −80 °C for the evaluation of malondialdehyde (MDA), fatty acid content, apelin, and RvD1.

### 4.2. Testosterone Evaluation in Blood Serum

The testosterone concentration in rabbit serum was performed by radioimmunoassay (RIA) using the Testosterone RIA KIT (Ref: RK-61M Institute of Isotopes Co. Ltd., Bucharest, Romania) as reported in Castellini et al. [2].

### 4.3. Semen Quality Assessment

Semen samples were collected by means of an artificial vagina heated to 38 °C with water and immediately transferred to the laboratory. Sperm quality was immediately evaluated on raw samples, as reported by Castellini et al. [2]. The concentration of sperm was evaluated with a Thoma-Zeiss chamber, and record sperm motility (%) and curvilinear velocity (VCL, µm/s) was evaluated by means of a computer assisted sperm analysis (CASA) system. 

After, semen samples were centrifuged for 15 min, and the sperm cells were divided into three aliquots. One aliquot was processed for immunocytochemistry, and the other two aliquots of 10^8^ spermatozoa/mL were stored at −80 °C for the evaluation of the oxidative status and fatty acid profile.

### 4.4. Oxidative Status of Testis and Sperm

Lipid peroxidation in the testis was assessed by the MDA level. Rabbit tissue samples were homogenized in a 0.04 M K^+^-phosphate buffer (pH 7.4) containing 0.01% BHT (1:5 *w*/*v*). The homogenate was deproteinized with acetonitrile (1:1) and then centrifuged at 3000× *g* for 1 min. The supernatants were used for MDA analysis after pre-column derivatization with 2,4-dinitrophenylhydrazine according to the method published by Shara et al. [50] with minor modifications. The samples were immediately stirred, extracted with 5 mL of pentane, and dried using nitrogen. MDA hydrazone was quantified by isocratic HPLC using a Waters 600 E system controller HPLC instrument equipped with a Waters Dual 2487 UV detector set at 307 nm. A 5 µm Ultrasphere ODS C18 column was used with a mobile phase composed of acetonitrile (45%) and HCl 0.01 N (55%) at a flow rate of 0.8 mL/min. A calibration curve with MDA concentrations ranging from 0.2 to 10 nmol/mL was used for quantification. The MDA concentration was calculated by peak areas using an Agilent 3395 integrator. The results are expressed as nmol/g tissue. 

The extent of sperm lipid peroxidation (thiobarbituric reactive substances, TBARs) was assessed by measuring malondialdehyde (MDA) along with other substances that are reactive to 2-thiobarbituric acid (TBA), as reported by Mourvaki et al. [51]. The results were expressed as nmol MDA/mL.

### 4.5. Fatty Acid Profiles of Diets, Testis and Sperm

Lipids were extracted from the sperm and testis according to Folch et al. [52]; the esterification was performed according to Christie [53]. The trans-methylation procedure was performed using eicosenoic acid methyl esters (Sigma-Aldrich, Bellefonte, PA, USA) as an internal standard. 

The fatty acid composition was determined using a Varian gas chromatograph (CP-3800) equipped with a flame ionization detector and a capillary column 100 m long × 0.25 mm × 0.2 μm film (Supelco, Bellefonte, PA, USA). Helium was used as the carrier gas with a flow of 0.6 mL/min. The split ratio was 1:20. The oven temperature was programmed, as reported by Mattioli et al. [54]. Individual fatty acid methyl esters (FAMEs) were detected by comparing the relative retention times of peaks in the sample with those of a standard mixture (FAME Mix Supelco; 4: 0 to 24: 0) plus cis- 395 9 cis-12 C18: 2; cis-9 cis-12 cis-15 C18: 3; and cis-9 cis-12 cis-15 C18: 3 (all from Sigma-Al- 396 drich). The fatty acids were expressed as % of total fatty acids. The average amount of each 397 fatty acid was used to calculate the sum of the total saturated fatty acid, monounsaturated 398 fatty acid, and polyunsaturated fatty acids (PUFA) n-3 and n-6. The very long-chain PUFA 399 (VLCP) included: EPAn-3, DPAn-3, and DHAn-3 acids, and arachidonic (ARAn-6) and 400 DPAn-6 were determined. The fatty acids profile of diets and registered feed intake was 401 used to estimate the daily intake of -3 PUFA and n-6 PUFA (g/day).

### 4.6. Immunofluorescence in Testis and Sperm

Small pieces of rabbit testes were fixed with 10% buffered formalin for 24 h at 4 °C, and then washed in water for 1 h. The samples were dehydrated in an increasing series of ethanol and cleared with xylene. Then, the specimens were incubated with three infiltrations of molten paraffin at 60 °C for 1 h, and solidified at room temperature. Paraffin sections were obtained by a Leica RM2125 RTS microtome (Leica Biosystem, Wetzlar, Germany); sections were deparaffinized with xylene, dehydrated in a series of ethanol concentrations for 5 min, and finally, in water. For antigen retrieval, the sections were washed and treated with heat-induced epitope retrieval 1 (HIER 1) buffer (10 mM sodium citrate) at pH 6 for 20 min at 95 °C. 

Sperm were smeared on slides, fixed in 4% paraformaldehyde for 15 min and treated with a blocking solution (phosphate-buffered saline (PBS)–bovine serum albumin (BSA) 1% Normal Goat Serum (NGS) 5%) for 20 min. 

Specimens were treated overnight at 4 °C with the rbbit anti-apelin polyclonal antibody (Abcam, Cambridge, UK) diluted 1:500 or rabbit anti-APJ-R polyclonal antibody (Thermo Fisher Scientific, Waltham, MA, USA) diluted 1:100 or anti-ryanodine receptor polyclonal antibody (Invitrogen, Thermo Fisher Scientific, Carlsbad, CA, USA) diluted 1:500. After three washes for 10 min in PBS, reactions were revealed by an anti-rabbit antibody raised in goat Alexa Fluor^®^ 488 conjugate (Invitrogen, Thermo Fisher Scientific, Carlsbad, CA, USA), diluted at 1:500 for 1 h at room temperature. Primary antibodies were omitted in the control slides. Nuclei were stained with 4,6-diamidino-2-phenylindole (DAPI) solution (Vysis, Downers Grove, IL, USA) for 10 min; the slides were washed in PBS and mounted with 1,4-diazabicyclo[2.2.2]octane (DABCO, Sigma-Aldrich, Milan, Italy) to observe fluorescence. All the samples were analyzed under a Leica DMI 6000 fluorescence microscope (Leica Microsystems, Wetzlar, Germany) with a 63× objective, and the images were acquired using the Leica AF 6500 Integrated System for imaging and analysis.

For standardization and comparison of the different groups, only good-quality sections at the same magnification were investigated; at least 10 sections of tissue from each group were evaluated. Two hundred sperm per sample were evaluated. In detail, the images were obtained with HCX PL FLUOTAR 63×/1.25 oil objective; filters for TRIC and FITC were selected. The micrographs were not modified with image elaboration software. The specificity of the antibodies, guaranteed in the datasheets of both antibodies, was also evaluated by omitting the primary antibodies

### 4.7. Resolvin (Rv) D1 and Apelin Assays in Testis

In homogenates of testicular tissue (75% *w*/*v*, in phosphate-buffered solution, pH 7.4), RvD1 and apelin were measured by a quantitative sandwich enzyme-linked immunosorbent assay (ELISA) (MBS2601295 MyBioSource, San Diego, CA, USA, and abx585113, Abbexa, Cambridge, UK, respectively). A biotin-labeled antibody and horseradish peroxidase + avidin were applied for bound biotin-labeled antibody detection. Spectrometric detection of color intensity at 450 nm allowed the determination of testis RvD1 and apelin amounts by comparing the optical density of each sample to the standard curve (respectively ranging from 2000 pg/mL to 31.2 pg/mL RvD1 amounts, ranging from 8000 to 125-pg/mL apelin amounts). In all experiments, measures were performed in duplicate in each sample.

### 4.8. Statistical Analysis

Lipid oxidation, fatty acids profile, testosterone, and sperm kinetic traits were analyzed with a linear model to evaluate the fixed effect of diet (control, FLAX, FISH; SPSS v28) Least squares (LS) mean, and pooled standard error (SE) are reported. The Bonferroni correction was applied for multiple comparisons. The significance was set at *p* < 0.05. A correlation was built to detect associations among the main variables (apelin, RvD1, MDA, n-3 PUFA, n-6 PUFA, final testosterone, MDA, sperm motility, curvilinear velocity VCL), also within dietary groups (Supplementary Table S1). The correlation was defined as high when the Pearson coefficient was (r) > |0.5|, medium when r ranged from 0.3 to 0.5, and low when r < |0.3|.

## 5. Conclusions

The data seems to indicate that dietary supplementation of FLAX increases the amount of apelin in the testis, whereas the RvD1 increased in both the supplemented diets. 

Apelin in ejaculated sperm was mainly localized in the tail, and its positive correlations with sperm motility and RvD1 level could suggest an involvement of these molecules in male reproduction and probably a role in the resolution of inflammatory status induced by dietary challenges (acute or chronic).

The proposed diet, by influencing apelin levels, could directly and indirectly, stimulate the resolution of reproductive inflammation.

Further studies on the biological system (in vivo and in vitro) challenged with pro-inflammatory or pro-oxidative molecules could be done to find a relationship with other derived molecules.

## Figures and Tables

**Table 1:** Serum testosterone (pg/mL) and sperm characteristics (progressive motility %, curvilinear velocity, VCL µm/sec, malondialdehyde, MDA nmol MDA/mL, fatty acids, and FA % of total FA) of rabbits fed experimental diets. Polyunsaturated fatty acids, PUFA; very long chain PUFA, VLCP.

Groups	Serum Testosterone (pg/mL)	Sperm Motility (%)	VCL (µm/sec)	MDA (nmol MDA/mL)	n-3 PUFA (% of Total FA)	n-6 PUFA (% of Total FA)	n-3 VLCP (% of Total FA)	n-6 VLCP (% of Total FA)
Control	3.63 b	62.13 a	184.7 ab	2.82 a	0.63 a	38.56 c	0.39 a	26.12 c
FLAX	4.60 c	76.31 b	236.5 b	14.79 b	4.25 b	22.45 b	2.83 b	18.31 a
FISH	2.82 a	77.27 b	226.9 b	20.64 c	13.12 c	19.06 a	12.62 c	23.62 b
SE	0.15	1.29	7.05	0.31	0.15	0.21	0.11	0.29
*p*-value	<0.001	<0.001	<0.001	<0.001	<0.001	<0.001	<0.001	<0.001

On the same column with different letters (a–c) means *p* < 0.001.

**Table 2:** Apelin (ng/g), resolvin (RvD1) (pg/g), malondialdehyde MDA (nmol/g), polyunsaturated fatty acids (PUFA), and vary long chain PUFA (VLCP %) in testes of rabbits fed experimental diets.

Groups	Apelin (ng/g)	RvD1 (pg/g)	MDA (nmol/g)	n-3 PUFA (%)	n-6 PUFA (%)	n-3 VLCP (%)	n-6 VLCP (%)
Control	1.187 a	111.578 a	34.755 a	2.305 a	38.695 b	0.078 a	26.525 b
FLAX	2.400 b	382.058 b	42.295 c	7.747 c	32.428 a	1.602 c	18.700 a
FISH	1.166 a	394.157 b	37.370 b	4.470 b	32.262 a	0.950 bc	17.930 a
SE	0.197	20.971	0.270	0.192	0.312	0.036	0.325
*p* value	<0.001	<0.001	<0.001	<0.001	<0.001	<0.001	<0.001

On the same column with different letters (a–c) means *p* < 0.001.

**Table 3:** Correlations (Pearson’s coefficient) between all considered variables. Apelin, resolvin (RvD1), malondialdehyde (MDA), polyunsaturated acids (PUFA) n-3, n-6, final testosterone (T), and curvilinear velocity (VCL).

	RvD1 Testis	MDA Testis	n-3 PUFA Testis	n-6 PUFA Testis	T	MDA Sperm	n-3 PUFA Sperm	n-6 PUFA Sperm	VCL	Sperm Motility	n-3 PUFA Intake	n-6 PUFA Intake
Apelin testis	0.360	0.838 **	0.798 **	−0.408	0.692 **	0.145	−00.210	−0.292	0.567 *	0.376	0.819 **	0.859 **
RvD1 testis		0.608 **	0.736 **	−0.947 **	−0.002	0.930 **	0.721 **	−0.960 **	0.776 **	0.949 **	0.724 **	0.248
MDA testis			0.964 **	−0.639 **	0.742 **	0.387	−0.033	−0.531 *	0.708 **	0.607 **	0.980 **	0.900 **
n-3 PUFA testis				−0.780 **	0.593 **	0.560 *	0.168	−0.684 **	0.828 **	0.718 **	0.990 **	0.799 **
n-6 PUFA testis					−0.029	−0.936 **	−0.730 **	0.975 **	−0.888 **	−0.945 **	−0.747 **	−0.267
T						−0.262	−0.621 **	0.105	0.192	−0.023	0.643 **	0.925 **
MDA sperm							0.904 **	−0.984 **	0.777 **	0.930 **	0.524 *	−0.031
n-3 PUFA sperm								−0.825 **	0.540 *	0.724 **	0.119	−0.448
n-6 PUFA sperm									−0.840 **	−0.956 **	−0.657 **	−0.133
VCL										0.815 **	0.779 **	0.406
Sperm motility											0.704 **	0.228
n-3 PUFA intake												0.406

* Correlation is significant at the 0.05 level (2-tailed). ** Correlation is significant at the 0.01 level (2-tailed).

**Table 4:** Formulation (g/kg of diet), chemical composition (g/kg of diet), and main fatty acids (% of total fatty acids) of diets. Linoleic acid, LA; alpha-linolenic acid, ALA; polyunsaturated acids, PUFA; long-chain PUFA, VLCP.

	Control	FLAX	FISH
Dehydrated alfalfa meal	300	380	380
Soybean meal 44%	150	100	150
Barley meal	410	310	335
Wheat bran	52	52	52
Soybean oil	30	-	-
Extruded flaxseed	-	100	-
Fish oil	-	-	35
Beet molasses	20	10	10
Calcium carbonate	7	7	7
Calcium diphosphate	13.5	13.5	13.5
Salt	7	7	7
DL-methionine	0.5	0.5	0.5
Vitamin-mineral premix †	10	10	10
Crude protein	175	174	175
Ether extract	480	472	425
Crude fiber	124	137	130
Ash	89	84	90
LA	50.45	22.30	20.50
ALA	11.15	45.80	18.50
n-6 PUFA	51.45	22.80	21.00
n-3 PUFA	11.35	46	26.40
n-3 VLCP	-	-	10.50

Nordic Naturals Omega-3^®^ = purified deep sea fish oil (from anchovies and sardines) containing EPA—330 mg/100 g, DHA—220 mg/100 g, and other VLCP—140 mg/100 g + α-tocopherol for preservation. † Per kg diet: vitamin A—11.000 IU; vitamin D_3_—2000 IU; vitamin B_1_—2.5 mg; vitamin B_2_—4 mg; vitamin B_6_—1.25 mg; vitamin B_12_—0.01 mg; alpha-tocopheryl acetate—200 mg; biotine—0.06 mg; vitamin K—2.5 mg; niacin—15 mg; folic acid—0.30 mg; D-pantothenic acid—10 mg; choline—600 mg; Mn—60 mg; Fe—50 mg; Zn—15 mg; I—0.5 mg; Co—0.5 mg.

## Data Availability

Not applicable.

## Supplementary Materials

The following supporting information can be downloaded at: https://www.mdpi.com/article/10.3390/molecules28176188/s1, Table S1: Correlations (Pearson’s coefficient) between all considered variables.
