# Supplementary material for: Can Dietary n-3 Polyunsaturated Fatty Acids Affect Apelin and Resolvin in Testis and Sperm of Male Rabbits?
Source: Molecules. 2023 Aug 22;28(17):6188. doi: 10.3390/molecules28176188 (PMC10488499; doi:10.3390/molecules28176188)
Supplement: Supplementary file 1 [file molecules-28-06188-s001.zip › molecules-2537602-supplementary.pdf]

**Table S1.** Correlations (Pearson's coefficient) between all considered variables. Apelin, resolvin (Rv) D1, malondialdehyde (MDA), polyunsaturated acids (PUFA) n-3, n-6 of testis and semen, final testosterone (T), thiobarbituric reactive substances (TBARS), curvilinear velocity (VCL), motiliy and PUFAn-3 and n-6 intake (g/d)

| | | Apelina testis | | | Resolvina testis | | | MDA testis | | | PUFAn-3 testis | | | PUFAn-6 testis | | | testosterone | | | TBARS sperm | | | PUFAn-3 sperm | | | PUFAn-6 sperm | | | VCL | | | motility | | | PUFAn-3 intake_g/d | | | PUFAn-6 intake_g/d | | |
|---|---|---|---|---|---|---|---|---|---|---|---|---|---|---|---|---|---|---|---|---|---|---|---|---|---|---|---|---|---|---|---|---|---|---|---|---|---|---|---|---|
| | | CNT | FLAX | FISH | CNT | FLAX | FISH | CNT | FLAX | FISH | CNT | FLAX | FISH | CNT | FLAX | FISH | CNT | FLAX | FISH | CNT | FLAX | FISH | CNT | FLAX | FISH | CNT | FLAX | FISH | CNT | FLAX | FISH | CNT | FLAX | FISH | CNT | FLAX | FISH | CNT | FLAX | FISH |
| Apelina testis | Corr | 1 | 1 | 1 | ,564 | -,847* | -,112 | -,138 | ,250 | -,503 | ,461 | -,028 | -,629 | -,727 | ,007 | ,464 | -,599 | ,139 | -,854* | -,046 | -,771 | -,398 | ,173 | ,688 | ,287 | ,685 | -,308 | ,691 | 0.655 | 0.715 | -,583 | 0.574 | -0.323 | -,227 | -0.380 | 0.397 | 0.697 | -0.380 | 0.397 | 0.697 |
| | Sign | | | | ,243 | ,034 | ,833 | ,794 | ,633 | ,309 | ,358 | ,958 | ,181 | ,102 | ,989 | ,355 | ,209 | ,793 | ,030 | ,931 | ,073 | ,435 | ,744 | ,131 | ,581 | ,133 | ,553 | ,129 | 0.158 | 0.111 | ,224 | 0.234 | 0.532 | ,665 | 0.458 | 0.436 | 0.124 | 0.458 | 0.436 | 0.124 |
| Resolvina testis | Corr | ,564 | -,847* | -,112 | 1 | 1 | 1 | ,158 | -,400 | -,256 | -,304 | -,475 | -,296 | -,707 | ,379 | ,118 | ,163 | -,506 | -,313 | -,646 | ,953** | -,237 | -,103 | -,818* | ,358 | ,282 | -,097 | ,482 | -0.169 | -,864* | -,568 | 0.346 | 0.496 | -,144 | 0.058 | -0.109 | 0.322 | 0.058 | -0.109 | 0.322 |
| | Sign | ,243 | ,034 | ,833 | | | | ,765 | ,432 | ,625 | ,558 | ,341 | ,570 | ,116 | ,458 | ,824 | ,758 | ,306 | ,546 | ,166 | ,003 | ,651 | ,845 | ,047 | ,486 | ,588 | ,855 | ,333 | 0.749 | 0.027 | ,239 | 0.502 | 0.317 | ,786 | 0.912 | 0.837 | 0.534 | 0.912 | 0.837 | 0.534 |
| MDA testis | Corr | -,138 | ,250 | -,503 | ,158 | -,400 | -,256 | 1 | 1 | 1 | ,034 | ,217 | ,029 | -,154 | -,376 | -,562 | ,012 | ,233 | ,496 | -,281 | -,205 | ,732 | -,080 | ,412 | -,479 | -,310 | ,197 | -,231 | -0.121 | 0.617 | ,281 | 0.220 | 0.152 | ,827* | ,926** | -0.273 | -0.662 | ,926** | -0.273 | -0.662 |
| | Sign | ,794 | ,633 | ,309 | ,765 | ,432 | ,625 | | | | ,949 | ,680 | ,957 | ,771 | ,463 | ,246 | ,982 | ,657 | ,317 | ,590 | ,696 | ,098 | ,880 | ,418 | ,337 | ,551 | ,708 | ,659 | 0.819 | 0.192 | ,590 | 0.675 | 0.774 | ,042 | 0.008 | 0.600 | 0.152 | 0.008 | 0.600 | 0.152 |
| PUFAn-3 testis | Corr | ,461 | -,028 | -,629 | -,304 | -,475 | -,296 | ,034 | ,217 | ,029 | 1 | 1 | 1 | -,146 | -,829* | ,167 | -,941** | ,569 | ,837* | ,201 | -,548 | ,392 | ,170 | ,521 | -,008 | ,672 | ,648 | -,697 | ,951** | 0.432 | ,541 | -0.007 | -0.601 | -,327 | -0.023 | -0.331 | -0.490 | -0.023 | -0.331 | -0.490 |
| | Sign | ,358 | ,958 | ,181 | ,558 | ,341 | ,570 | ,949 | ,680 | ,957 | | | | ,783 | ,041 | ,752 | ,005 | ,238 | ,037 | ,702 | ,260 | ,443 | ,748 | ,290 | ,988 | ,143 | ,164 | ,124 | 0.003 | 0.392 | ,268 | 0.989 | 0.207 | ,528 | 0.965 | 0.521 | 0.323 | 0.965 | 0.521 | 0.323 |
| PUFAn-6 testis | Corr | -,727 | ,007 | ,464 | -,707 | ,379 | ,118 | -,154 | -,376 | -,562 | -,146 | -,829* | ,167 | 1 | 1 | 1 | ,108 | -,086 | -,319 | ,514 | ,492 | ,048 | ,491 | -,363 | ,794 | -,415 | -,217 | ,489 | -0.340 | -0.259 | -,629 | -0.355 | 0.264 | -,729 | 0.053 | -0.073 | 0.285 | 0.053 | -0.073 | 0.285 |
| | Sign | ,102 | ,989 | ,355 | ,116 | ,458 | ,824 | ,771 | ,463 | ,246 | ,783 | ,041 | ,752 | | | | ,839 | ,872 | ,538 | ,297 | ,321 | ,928 | ,322 | ,479 | ,059 | ,413 | ,680 | ,325 | 0.509 | 0.620 | ,181 | 0.490 | 0.613 | ,100 | 0.920 | 0.891 | 0.583 | 0.920 | 0.891 | 0.583 |
| testosterone | Corr | -,599 | ,139 | -,854* | ,163 | -,506 | -,313 | ,012 | ,233 | ,496 | -,941** | ,569 | ,837* | ,108 | -,086 | -,319 | 1 | 1 | 1 | -,299 | -,400 | ,594 | -,454 | ,480 | -,439 | -,697 | ,804 | -,782 | -,913* | 0.621 | ,706 | -0.196 | -0.490 | ,224 | 0.107 | -0.720 | -0.644 | 0.107 | -0.720 | -0.644 |
| | Sign | ,209 | ,793 | ,030 | ,758 | ,306 | ,546 | ,982 | ,657 | ,317 | ,005 | ,238 | ,037 | ,839 | ,872 | ,538 | | | | ,565 | ,432 | ,214 | ,366 | ,335 | ,384 | ,124 | ,054 | ,066 | 0.011 | 0.189 | ,117 | 0.710 | 0.323 | ,670 | 0.840 | 0.106 | 0.168 | 0.840 | 0.106 | 0.168 |
| TBARS | Corr | -,046 | -,771 | -,398 | -,646 | ,953** | -,237 | -,281 | -,205 | ,732 | ,201 | -,548 | ,392 | ,514 | ,492 | ,048 | -,299 | -,400 | ,594 | 1 | 1 | 1 | ,584 | -,703 | -,186 | -,299 | ,001 | -,052 | 0.225 | -0.688 | ,004 | 0.407 | 0.490 | ,462 | -0.421 | -0.279 | -0.483 | -0.421 | -0.279 | -0.483 |
| | Sign | ,931 | ,073 | ,435 | ,166 | ,003 | ,651 | ,590 | ,696 | ,098 | ,702 | ,260 | ,443 | ,297 | ,321 | ,928 | ,565 | ,432 | ,214 | | | | ,223 | ,119 | ,725 | ,564 | ,998 | ,922 | 0.668 | 0.131 | ,993 | 0.423 | 0.324 | ,356 | 0.406 | 0.592 | 0.331 | 0.406 | 0.592 | 0.331 |
| PUFAn-3 sperm | Corr | ,173 | ,688 | ,287 | -,103 | -,818* | ,358 | -,080 | ,412 | -,479 | ,170 | ,521 | -,008 | ,491 | -,363 | ,794 | -,454 | ,480 | -,439 | ,584 | -,703 | -,186 | 1 | 1 | 1 | ,060 | ,368 | ,418 | 0.107 | ,928** | -,596 | 0.402 | -0.786 | -,761 | -0.126 | -0.170 | 0.002 | -0.126 | -0.170 | 0.002 |
| | Sign | ,744 | ,131 | ,581 | ,845 | ,047 | ,486 | ,880 | ,418 | ,337 | ,748 | ,290 | ,988 | ,322 | ,479 | ,059 | ,366 | ,335 | ,384 | ,223 | ,119 | ,725 | | | | ,910 | ,472 | ,409 | 0.841 | 0.008 | ,211 | 0.430 | 0.064 | ,079 | 0.811 | 0.747 | 0.997 | 0.811 | 0.747 | 0.997 |
| PUFAn-6 sperm | Corr | ,685 | -,308 | ,691 | ,282 | -,097 | ,482 | -,310 | ,197 | -,231 | ,672 | ,648 | -,697 | -,415 | -,217 | ,489 | -,697 | ,804 | -,782 | -,299 | ,001 | -,052 | ,060 | ,368 | ,418 | 1 | 1 | 1 | 0.720 | 0.388 | -,968** | -0.169 | -0.543 | -,017 | -0.275 | -,921** | 0.624 | -0.275 | -,921** | 0.624 |
| | Sign | ,133 | ,553 | ,129 | ,588 | ,855 | ,333 | ,551 | ,708 | ,659 | ,143 | ,164 | ,124 | ,413 | ,680 | ,325 | ,124 | ,054 | ,066 | ,564 | ,998 | ,922 | ,910 | ,472 | ,409 | | | | 0.107 | 0.447 | ,002 | 0.748 | 0.265 | ,974 | 0.598 | 0.009 | 0.186 | 0.598 | 0.009 | 0.186 |
| VCL | Corr | 0.655 | 0.715 | -,583 | -0.169 | -,864* | -,568 | -0.121 | 0.617 | ,281 | ,951** | 0.432 | ,541 | -0.340 | -0.259 | -,629 | -,913* | 0.621 | ,706 | 0.225 | -0.688 | ,004 | 0.107 | ,928** | -,596 | 0.720 | 0.388 | -,968** | 1 | 1 | 1 | 0.149 | -0.569 | ,180 | -0.252 | -0.282 | -0.523 | -0.252 | -0.282 | -0.523 |
| | Sign | 0.158 | 0.111 | ,224 | 0.749 | 0.027 | ,239 | 0.819 | 0.192 | ,590 | 0.003 | 0.392 | ,268 | 0.509 | 0.620 | ,181 | 0.011 | 0.189 | ,117 | 0.668 | 0.131 | ,993 | 0.841 | 0.008 | ,211 | 0.107 | 0.447 | ,002 | | | | 0.778 | 0.239 | ,733 | 0.630 | 0.588 | 0.287 | 0.630 | 0.588 | 0.287 |
| motility | Corr | 0.574 | -0.323 | -,227 | 0.346 | 0.496 | -,144 | 0.220 | 0.152 | ,827* | -0.007 | -0.601 | -,327 | -0.355 | 0.264 | -,729 | -0.196 | -0.490 | ,224 | 0.407 | 0.490 | ,462 | 0.402 | -0.786 | -,761 | -0.169 | -0.543 | -,017 | 0.149 | -0.569 | ,180 | 1 | 1 | 1 | -0.137 | 0.271 | -0.172 | -0.137 | 0.271 | -0.172 |
| | Sign | 0.234 | 0.532 | ,665 | 0.502 | 0.317 | ,786 | 0.675 | 0.774 | ,042 | 0.989 | 0.207 | ,528 | 0.490 | 0.613 | ,100 | 0.710 | 0.323 | ,670 | 0.423 | 0.324 | ,356 | 0.430 | 0.064 | ,079 | 0.748 | 0.265 | ,974 | 0.778 | 0.239 | ,733 | | | | 0.796 | 0.603 | 0.745 | 0.796 | 0.603 | 0.745 |
| PUFAn-3 intake_g/d | Corr | -0.380 | 0.397 | 0.697 | 0.058 | -0.109 | 0.322 | ,926** | -0.273 | -0.662 | -0.023 | -0.331 | -0.490 | 0.053 | -0.073 | 0.285 | 0.107 | -0.720 | -0.644 | -0.421 | -0.279 | -0.483 | -0.126 | -0.170 | 0.002 | -0.275 | -,921** | 0.624 | -0.252 | -0.282 | -0.523 | -0.137 | 0.271 | -0.172 | 1 | 1 | 1 | 1,000** | 1,000** | 1,000** |
| | Sign | 0.458 | 0.436 | 0.124 | 0.912 | 0.837 | 0.534 | 0.008 | 0.600 | 0.152 | 0.965 | 0.521 | 0.323 | 0.920 | 0.891 | 0.583 | 0.840 | 0.106 | 0.168 | 0.406 | 0.592 | 0.331 | 0.811 | 0.747 | 0.997 | 0.598 | 0.009 | 0.186 | 0.630 | 0.588 | 0.287 | 0.796 | 0.603 | 0.745 | | | | 0.000 | 0.000 | 0.000 |
| PUFAn-6 intake_g/d | Corr | -0.380 | 0.397 | 0.697 | 0.058 | -0.109 | 0.322 | ,926** | -0.273 | -0.662 | -0.023 | -0.331 | -0.490 | 0.053 | -0.073 | 0.285 | 0.107 | -0.720 | -0.644 | -0.421 | -0.279 | -0.483 | -0.126 | -0.170 | 0.002 | -0.275 | -,921** | 0.624 | -0.252 | -0.282 | -0.523 | -0.137 | 0.271 | -0.172 | 1,000** | 1,000** | 1,000** | 1 | 1 | 1 |
| | Sign | 0.458 | 0.436 | 0.124 | 0.912 | 0.837 | 0.534 | 0.008 | 0.600 | 0.152 | 0.965 | 0.521 | 0.323 | 0.920 | 0.891 | 0.583 | 0.840 | 0.106 | 0.168 | 0.406 | 0.592 | 0.331 | 0.811 | 0.747 | 0.997 | 0.598 | 0.009 | 0.186 | 0.630 | 0.588 | 0.287 | 0.796 | 0.603 | 0.745 | 0.000 | 0.000 | 0.000 | | | |

*. Correlation is significant at the 0.05 level (2-tailed).

**. Correlation is significant at the 0.01 level (2-tailed).
